# Beyond the Scalpel: Addressing Communication and Distress in ENT Cancer

**DOI:** 10.3390/medicina61010069

**Published:** 2025-01-03

**Authors:** Bogdan Hirtie, Norberth-Istvan Varga, Andrada Oprisoni, Sonia Tanasescu, Estera Boeriu, Virgiliu Bogdan Sorop, Octavia Harich, Adrian Vasile Bota, Delia Ioana Horhat

**Affiliations:** 1 Doctoral School, “Victor Babes” University of Medicine and Pharmacy, 300041 Timisoara, Romania; bogdan.hirtie@umft.ro (B.H.); norberth.varga@umft.ro (N.-I.V.); 2 Department of Pediatrics, Discipline of Pediatric Oncology and Hematology, “Victor Babes” University of Medicine and Pharmacy, 300041 Timisoara, Romania; oprisoni.licinia@umft.ro; 3 Department of Pediatrics, “Victor Babes” University of Medicine and Pharmacy, 300041 Timisoara, Romania; tanasescu.sonia@umft.ro; 4 Department of Obstetrics and Gynecology, “Victor Babes” University of Medicine and Pharmacy, 300041 Timisoara, Romania; bogdan.sorop@umft.ro; 5 Department of Functional Sciences, “Victor Babes” University of Medicine and Pharmacy Timisoara, 300041 Timisoara, Romania; harich.octavia@umft.ro; 6 Multidisciplinary Doctoral School, “Vasile Goldis” Western University, 310419 Arad, Romania; bota.adrian-vasile@student.uvvg.ro; 7 Department of ENT, “Victor Babes” University of Medicine and Pharmacy, 300041 Timisoara, Romania; horhat.ioana@umft.ro

**Keywords:** communication preferences, questionnaire, ENT cancer, psychological distress, KOPRA, HADS, Hospital Anxiety and Depression Scale

## Abstract

*Background and Objectives*: Effective communication in oncology is crucial, but challenging due to the complex information and emotional burden associated with a cancer diagnosis. This cross-sectional study investigated the communication preferences of 155 Romanian adults diagnosed with ENT cancers and explored the relationship between these preferences, their levels of psychological distress, and sociodemographic factors. *Materials and Methods*: Participants completed the KOPRA questionnaire, assessing communication preferences, and the Hospital Anxiety and Depression Scale (HADS) to measure psychological distress. *Results*: The results revealed that patients strongly prioritized active involvement in their care (Patient Participation and Patient Orientation—PPO) and open communication with healthcare providers (Effective and Open Communication—EOC). While emotional support was valued, it was considered less critical than PPO and EOC. Notably, communication about personal matters was deemed the least important aspect of communication. A high prevalence of psychological distress was observed, particularly among widowed individuals and females. No direct correlation was found between communication preferences and distress. *Conclusions*: These findings underscore the importance of shared decision-making, clear information exchange, and a patient-centered approach in the context of ENT cancer care, while also highlighting the need for routine screening and appropriate support for psychological well-being in this patient population.

## 1. Introduction

Effective communication is a cornerstone of quality cancer care, playing a crucial role in patient satisfaction, treatment adherence, and overall well-being [1]. However, communication in oncology settings presents unique challenges. Cancer patients often face complex medical information and a significant emotional burden, all of which necessitate clear, empathetic, and individualized communication from healthcare providers [2,3]. When communication is successful, it can empower patients to actively participate in their care, cope with their illness, and maintain hope [4]. Conversely, poor communication can lead to confusion, anxiety, and diminished trust in the patient–provider relationship [5].

In recent years, there has been a growing recognition of the importance of patient-centered communication in cancer care [1,2,3,4,5,6,7]. This approach emphasizes active listening, empathy, and shared decision-making, where patients are viewed as partners in their care rather than passive recipients of treatment [8]. Several studies have investigated the communication preferences of cancer patients in various contexts, including during initial consultations, treatment discussions, and the delivery of bad news. Vicente et al. (2023) [9] examined patient preferences and perceptions during their first medical oncology appointment, finding that patients highly valued the physician’s knowledge, honesty, and ability to provide clear information. Kim et al. (2020) [10] explored patient preferences when receiving a cancer diagnosis, noting differences based on cancer stage and the desire for clear explanations and family involvement. Krieger et al. [11] also investigated patient experiences and preferences when receiving bad news, highlighting the importance of clear communication, a supportive environment, and opportunities for questions and comprehensive support.

Furthermore, research has shown that patients’ communication preferences may be influenced by factors such as age, gender, cultural background, and disease stage. For instance, studies have reported that younger patients may prefer more open and direct communication, while older patients may value a more traditional, paternalistic approach. Daskivich et al. [12] found that men with prostate cancer preferred specific, quantitative information about life expectancy rather than generalizations, contrasting with preferences observed in screening populations. This highlights the importance of considering the specific context and needs of different patient populations.

Additionally, research has explored the role of communication in addressing psychological distress among cancer patients. Back et al. [13] reviewed communication issues in palliative care, emphasizing the need for patient-centered approaches that address informational, emotional, and decision-making needs. Ryan et al. [14] assessed interventions to improve end-of-life communication, highlighting the complexity of such interactions and the need for further research. Ramchandani et al. [15] analyzed prognostic communication preferences in advanced cancer, finding that a substantial proportion of patients preferred to know their prognosis, influenced by their psychological state.

The KOPRA questionnaire (“Kommunikations-Präferenzen von Patienten mit chronischen Erkrankungen” or “Communication Preferences of Patients with Chronic Illness”) [16], developed by Farin et al. (2011), is a validated instrument designed to assess patient communication preferences in the context of chronic illness [16,17,18]. It comprises 32 items categorized into four distinct subscales: Patient Participation and Patient Orientation (PPO), Effective and Open Communication (EOC), Emotionally Supportive Communication (ESC), and Communication about Personal Circumstances (CPC). The KOPRA allows for a nuanced understanding of individual preferences, enabling healthcare providers to tailor their communication style and foster a more patient-centered approach to care.

Psychological distress, including anxiety and depression, is common among cancer patients and can significantly impact their quality of life and treatment outcomes. The Hospital Anxiety and Depression Scale (HADS) is a widely used and validated instrument for assessing the severity of anxiety and depression symptoms in medical populations [19]. Given the emotional burden associated with a cancer diagnosis and treatment, it is essential to consider the psychological well-being of patients. The HADS has demonstrated sensitivity in detecting clinically significant levels of anxiety and depression [20,21]. By incorporating the HADS into this study, we aim to gain a comprehensive understanding of the psychological state of ENT (ears, nose, and throat) cancer patients and its potential relationship with their communication preferences.

This study aimed to investigate the communication preferences of patients diagnosed with ENT cancers and to explore the relationship between these preferences and their levels of psychological distress. By examining these factors, this study sought to contribute to a deeper understanding of patient-centered communication in the context of ENT cancer care and to inform strategies for enhancing patient–provider interactions and improving patient well-being.

## 2. Materials and Methods

### 2.1. Participants and Procedure

This cross-sectional study was conducted within the Department of Otolaryngology (ENT) at the County Hospital of Timisoara, Romania, between 1 February and 30 September 2024. This study focused on adult patients (over 18 years old) diagnosed with various cancers within the ENT sphere, including larynx, rhino-pharynx, tongue base, and vocal cord cancers. Eligibility for participation required a confirmed cancer diagnosis, the provision of informed consent, and the mental and psychological capacity to engage in the study. Patients under 18, those with cognitive impairments, and those who declined participation were excluded.

Data collection occurred over a timespan of eight months, from 1 February to 30 September 2024. During this period, eligible patients were approached bedside during their hospitalization for treatment (radiotherapy, chemotherapy, and/or surgery) or at subsequent follow-up appointments. They were provided with the KOPRA questionnaire and the Hospital Anxiety and Depression Scale (HADS) to assess their communication preferences and levels of anxiety and depression, respectively. The questionnaires were administered in person at the hospital during these visits. We provided clear and concise instructions to participants, in order to minimize any misunderstanding or bias in their responses. Demographic information (age, sex, marital status, area of residence, occupation, and qualifications) and clinical variables (tumor location, disease status, current treatment approach, and time since diagnosis) were also gathered. To ensure ethical conduct, this study was performed in accordance with the Declaration of Helsinki. To maintain patient confidentiality, only the authors involved in data collection had access to the patients’ full names. The remaining authors, who performed the statistical analyses and wrote the manuscript, worked with de-identified data using only initials. This study received approval from the Ethics Committee of the Timisoara County Hospital (protocol code 2402/15 April 2024).

### 2.2. The KOPRA and HADS Questionnaires–Measures and Translation

The KOPRA questionnaire comprises 32 items categorized into four distinct subscales: Patient Participation and Patient Orientation (PPO), Effective and Open Communication (EOC), Emotionally Supportive Communication (ESC), and Communication about Personal Circumstances (CPC). The PPO subscale focuses on the patient’s desire for active involvement in decision-making and a patient-centered approach. The EOC subscale assesses the preference for clear, comprehensive, and honest communication. The ESC subscale evaluates the importance of emotional support, empathy, and a warm interpersonal connection. Lastly, the CPC subscale explores the patient’s desire for discussions about personal matters and establishing a deeper relationship with their healthcare provider. By utilizing a 5-point Likert scale, ranging from “not important at all” to “extremely important”, the KOPRA questionnaire allows for a nuanced understanding of individual preferences, enabling healthcare providers to tailor their communication style and foster a more patient-centered approach to care.

The HADS is a 14-item self-report questionnaire with two subscales, one for anxiety and one for depression. Each item is scored on a 4-point Likert scale (0–3), with higher scores indicating greater distress. A total score of 0–7 on each subscale is considered within the normal range, 8–10 indicates borderline abnormal, and 11–21 indicates abnormal.

The KOPRA questionnaire, originally developed in German, was translated into Romanian for this study. The translation process involved two independent translators fluent in both languages, who produced separate forward translations. These translations were then reconciled to resolve any discrepancies, resulting in a single Romanian version. This version was back-translated into German by a third independent translator, blind to the original scale. An expert panel, including the original scale developer, compared the back-translation to the original German scale to ensure conceptual equivalence. The final Romanian version was then finalized based on this review.

### 2.3. Statistical Analysis

Descriptive statistics were employed to summarize the demographic and clinical characteristics of the participants. Cronbach’s alpha was calculated to assess the internal consistency of the KOPRA subscales. Pearson correlation coefficients were used to examine the relationship between age and the HADS depression and anxiety scores. Independent samples *t*-tests were conducted to compare HADS depression and anxiety scores between males and females. ANOVAs were performed to examine the effect of marital status on depression and anxiety scores, followed by post hoc tests (Tukey’s HSD) to identify specific group differences. To examine the influence of sociodemographic factors on communication preferences, multiple linear regression analyses were conducted with each KOPRA subscale as the dependent variable. The normality of residuals was assessed using histograms and P-P plots to ensure the validity of the regression results. A significance level of *p* < 0.05 was used for all statistical tests. Data were analyzed using SPSS version 26.

## 3. Results

### 3.1. Patient Demographics

During the data collection period, 206 questionnaires were given to patients who met the inclusion criteria, and 19 patients refused to participate in the study, resulting in an acceptance rate of 90.77%. Of the 187 questionnaires distributed to consenting patients, 32 were excluded due to incomplete data, resulting in a final sample of 155 fully completed questionnaires. The age of participants ranged from 18 to 83, with a mean age of 64.29 years (standard deviation = 10.9). The most common diagnoses were laryngeal, pharyngeal, vocal cord, and tongue base cancers. In total, 74.83% of patients (*n* = 116) had received their diagnosis within the past three months, with an average duration since diagnosis of 4.5 months. All patients were actively undergoing some form of treatment (e.g., radiotherapy, chemotherapy, and/or surgery) at the time they completed the HADS and the KOPRA questionnaire.

Regarding marital status, 44.5% were married or cohabiting, 21.3% were divorced, 20% were single, and 13.5% were widowed. In terms of occupation, 37.4% were employed, 45.2% were retired, 10.3% were on welfare, and 7.1% were unemployed. All participants were Romanian citizens. Table 1 provides a comprehensive overview of the demographic and medical characteristics of the study participants.

### 3.2. Communication Preferences According to the KOPRA Questionnaire

The high Cronbach’s alpha values obtained for each subscale—0.919 for Patient Participation and Patient Orientation (PPO), 0.932 for Effective and Open Communication (EOC), 0.813 for Emotionally Supportive Communication (ESC), and 0.844 for Communication about Personal Circumstances (CPC)—affirm the robust internal consistency and reliability of these measures in capturing distinct facets of patient communication preferences.

The results of the KOPRA questionnaire indicate that cancer patients in this study placed the highest value on Patient Participation and Patient Orientation, with a mean score of 4.76. This suggests a strong preference for being actively involved in their treatment decisions and receiving care that is tailored to their individual needs and concerns. The second-highest-rated subscale was Effective and Open Communication (mean = 4.24), highlighting the importance of clear, comprehensive, and honest communication from healthcare providers. Emotionally Supportive Communication was considered moderately important (mean = 3.18), while Communication about Personal Circumstances was deemed the least important aspect of communication (mean = 2.15). These findings suggest that while patients value emotional support and a personal connection with their providers, they prioritize clear information and active involvement in their care. Table 2 provides insights into the communication preferences of cancer patients as assessed by the KOPRA questionnaire.

### 3.3. Patient Psychological Distress

This study assessed anxiety and depression levels in cancer patients using the Hospital Anxiety and Depression Scale (HADS). The mean score of the depression subscale of the HADS was 9.19 (SD = 2.7), indicating a moderate level of depression, while the mean score of the anxiety subscale was 10.28 (SD = 2.8), suggesting a moderate-to-high level of anxiety. As illustrated in Table 3, 105 (67.74%) patients exhibited a depression score of 8 or higher, indicating clinically significant depressive symptoms, while 118 (76.12%) reported moderate to severe anxiety symptoms, as evidenced by an anxiety score of 8 of higher.

Looking at the individual subscales of the HADS, 56 (36.12%) participants exhibited borderline abnormal levels of depression, with scores between 8 and 10, while 49 (31.61%) had scores equal to or greater than 11, indicating high levels of depression. In total, 48 (30.96%) participants exhibited borderline abnormal levels of anxiety, indicated by scores between 8 and 10, while 70 (45.16%) had scores greater than 11, meaning high levels of anxiety.

### 3.4. Influence of Depression and Anxiety on Communication Preferences

The normality of distributions was assessed using the Shapiro–Wilk test. As the assumption of normality was violated for all KOPRA subscales (all *p* < 0.05), a Kruskal–Wallis test was conducted to compare the KOPRA subscale scores between patients with and without clinically significant depressive symptoms (with HADS—depression score >8). No significant differences were found between the groups for any of the KOPRA subscales: PPO (*p* = 0.381), EOC (*p* = 0.705), ESC (*p* = 0.475), and CPC (*p* = 0.256). These results suggest that communication preferences, as measured by the KOPRA questionnaire, do not differ significantly based on the presence or absence of depressive symptoms in ENT cancer patients. Similarly, a Kruskal–Wallis test was conducted to compare the KOPRA subscale scores between patients with and without clinically significant anxiety symptoms (with HADS—anxiety score > 8). No significant differences were found between the groups for any of the KOPRA subscales: PPO (*p* = 0.323), EOC (*p* = 0.622), ESC (*p* = 0.815), and CPC (*p* = 0.266). These results further suggest that communication preferences, as measured by the KOPRA, do not differ significantly based on the presence or absence of anxiety symptoms in ENT cancer patients.

### 3.5. Sociodemographic Predictors of Psychological Distress

Pearson correlations were conducted to examine the relationship between age and psychological distress. No significant correlations were found between age and depression scores (r = 0.135, *p* = 0.095) or between age and anxiety scores (r = 0.057, *p* = 0.480).

Independent samples *t*-tests were conducted to compare HADS depression and anxiety scores between males and females. There was a marginally significant difference in depression scores (*p* = 0.055), with females having slightly higher scores (mean = 10.11 ± 4.06) than males (mean = 8.80 ± 3.18). There was a statistically significant difference in anxiety scores (*p* = 0.045), with females having significantly higher scores (mean = 11.22, ± 4.21) than males (mean = 9.88, ± 3.55).

ANOVAs were performed to examine the effect of marital status on depression and anxiety scores, revealing significant differences in both depression scores (*p* = 0.037) and anxiety scores (*p* = 0.036) among patients of different marital statuses. Post hoc tests using Tukey’s HSD showed that widowed individuals had significantly higher depression scores (mean = 11.10 ± 2.19) compared to those who were married or living with a partner (mean = 8.74 ± 2.58). Similarly, widowed individuals had significantly higher anxiety scores (mean = 12.10 ± 3.41) compared to both divorced individuals (mean = 9.67 ± 3.91) and those who were married or living with a partner (mean = 9.74 ± 3.27). These findings are illustrated in Table 4.

### 3.6. Communication Preferences Based on Sociodemographic Factors

To examine the influence of sociodemographic factors on communication preferences, a series of linear regression analyses were conducted with each KOPRA subscale as the dependent variable. The impact of a broad range of sociodemographic factors (age, sex, area of residence (urban/rural), marital status, qualifications, and occupation) was assessed for each KOPRA subscale.

The regression model for PPO explained 7.6% of the variance (R^2^ = 0.076, *p* = 0.006). The only significant predictor in the model was sex (β = 0.333, *p* = 0.002), indicating that females had a stronger preference for PPO than males. Age and other sociodemographic factors were not significantly associated with PPO preferences. The regression model for EOC explained 9.7% of the variance (R^2^ = 0.097, *p* = 0.044). Similar to PPO, sex was the only significant predictor (β = 0.336, *p* = 0.005), with females demonstrating a stronger preference for EOC compared to males. Other sociodemographic factors were not significantly associated with EOC preferences.

The regression model for ESC demonstrated negligible explanatory power (R^2^ = 0.030, *p* = 0.688). None of the sociodemographic factors emerged as significant predictors of ESC preferences. The regression model for CPC explained 14.4% of the variance (R^2^ = 0.144, *p* = 0.003). Two sociodemographic factors emerged as significant predictors: age (β = −0.006, *p* = 0.002) and area of residence (β = 0.100, *p* = 0.012). Older patients tended to place less importance on CPC, while patients from rural areas had a stronger preference for CPC. The normality of residuals was confirmed for all regression models.

## 4. Discussion

This study aimed to investigate the communication preferences of patients diagnosed with ENT cancers and explore the relationship between these preferences and their levels of psychological distress. Our analysis revealed key insights into the communication needs and psychological well-being of this patient population.

### 4.1. Communication Preferences

The analysis revealed that patients strongly prioritize active involvement in their care and open communication with their healthcare providers. Specifically, patients placed high importance on Patient Participation and Patient Orientation (PPO) and Effective and Open Communication (EOC), as evidenced by the high mean scores on the KOPRA questionnaire. While Emotionally Supportive Communication (ESC) was also valued, it was considered less critical than PPO and EOC. Interestingly, Communication about Personal Circumstances (CPC) emerged as the least important aspect of communication for these patients. These findings underscore the importance of shared decision-making, clear information exchange, and a patient-centered approach in the context of ENT cancer care. A study from Ullrich et al. (2014) found that patients with fibromyalgia syndrome also prioritized PPO and EOC, similarly to the ENT cancer patients in our study [17]. This suggests a consistent pattern of preferences across different chronic illnesses, where patients value clear information, shared decision-making, and active involvement in their care. Furthermore, both studies found that Communication about Personal Circumstances was the least important aspect of communication. This suggests that, while patients with chronic illnesses value a supportive and empathetic relationship with their healthcare providers, they prioritize instrumental aspects of communication, such as receiving clear information and participating in decision-making, over personal discussions.

Consistent with previous research, females in our study demonstrated a stronger preference for patient-centered and open communication. The observed stronger preference for PPO and EOC among female patients is consistent with previous studies indicating that women tend to elicit and prefer more patient-centered and participatory communication styles in medical interactions [22,23]. However, this contrasts with the findings of McKinstry (2000), who observed no major association between sex and preference for shared decision-making in a general practice setting [24]. This discrepancy might be explained by the specific context of cancer care, where patients may be more likely to seek active involvement and open communication due to the high stakes and emotional burden associated with the illness [25,26].

In our study, age was a significant predictor of patients’ desire for communication about personal circumstances (CPC). Older patients tend to place less importance on CPC, while younger patients may actively seek a more personal connection with their healthcare providers. These findings align with studies that suggest that older adults may prefer a more task-oriented and less personal approach to communication [27,28]. However, it is important for healthcare providers to recognize that this is not a universal preference and to remain open to discussing personal matters with older patients who express a desire for such communication. Similarly, Yoshida et al. (2023) [29] explored the communication preferences of adolescent and young adult (AYA) patients receiving a cancer diagnosis, revealing the importance of considering age-specific needs and preferences, such as addressing generation-specific social factors and supporting their decision-making in a developmentally appropriate manner.

Area of residence also influenced CPC preferences, with patients from rural areas showing a stronger preference for discussing personal matters with their healthcare providers. This could be attributed to factors such as limited access to healthcare resources in rural areas or a stronger sense of community that fosters closer patient–provider relationships. The findings of Gu et al. (2019) [30] support this notion, as they observed that in rural Chinese village clinics, patient–doctor trust was indirectly influenced by contract service policies aimed at improving healthcare access, with the perceived quality of doctor communication acting as a mediator. This suggests that in settings with potentially limited healthcare resources, the quality of communication and the personal connection with healthcare providers may be particularly valued by patients, potentially explaining the increased emphasis on CPC among rural patients in our study.

### 4.2. Psychological Distress

This study revealed a significant prevalence of psychological distress among the participants, with a majority reporting symptoms of depression and anxiety. This highlights the need for routine screening and appropriate support for psychological well-being in this patient population. Our findings regarding the prevalence of psychological distress among ENT cancer patients align with previous research highlighting the significant psychological burden associated with a cancer diagnosis. For instance, Razavi et al. (1990), using the HADS in a sample of head and neck cancer patients, found a similarly high prevalence of anxiety and depression symptoms [21]. The strong positive correlation we observed between anxiety and depression scores is consistent with findings from other studies demonstrating the frequent co-occurrence of these conditions in cancer patients [20]. Eadie et al. (2014) reported a significant prevalence of psychological distress among head-and-neck cancer patients, highlighting the importance of addressing mental health needs in this population [31]. Similarly, a study from Sauder et al. (2020) observed that pre-treatment oral and oropharyngeal cancer patients reported communication difficulties, which have been linked to depression and anxiety [32].

Notably, we found that widowed individuals experienced significantly higher levels of depression and anxiety compared to those who are married or cohabiting. This could be due to a number of factors, including the loss of a primary source of emotional and social support, increased financial strain, and the challenges of coping with grief and loneliness. These findings highlight the importance of providing targeted support to widowed patients, who may be particularly vulnerable to psychological distress. Additionally, there was a marginally significant difference in depression scores and a statistically significant difference in anxiety scores between males and females, with females exhibiting greater distress.

Despite the high prevalence of psychological distress in our sample, we found no significant direct correlations between the KOPRA subscales and HADS scores for depression and anxiety. The cross-sectional design limits the ability to establish causal relationships between communication preferences and distress. Longitudinal studies could provide more insights into how these factors interact over time. The KOPRA questionnaire, while comprehensive, might not capture all aspects of communication relevant to psychological well-being. Future research could explore other communication dimensions, such as patients’ perceptions of empathy, trust, and the quality of the patient–provider relationship.

### 4.3. Limitations

This study, while offering valuable insights into the communication preferences and psychological distress of ENT cancer patients, is not without limitations.

Primarily, the specific focus on ENT cancers, and, therefore, the relatively small sample size, may limit the generalizability of the findings to other cancer populations or healthcare settings.

Additionally, the reliance on self-report measures, while common in this field of research, introduces the possibility of response bias and common method bias. To mitigate the potential for common method bias, several measures were implemented. First, all patients responded to the questionnaires in person at the hospital, ensuring a standardized environment for data collection. Second, clear instructions were provided to participants to minimize any misunderstanding or bias in their responses. Third, to maintain patient confidentiality, only the authors involved in data collection had access to the patients’ full names. The remaining authors, who performed the statistical analyses and wrote the manuscript, worked with de-identified data using only initials.

The cross-sectional nature of this study precludes the establishment of causal relationships between communication preferences and psychological distress, warranting further longitudinal research to explore these dynamics over time. Furthermore, although the overall sample size was adequate, certain subgroups within the sample might have been underrepresented, potentially limiting the power of analyses within those groups.

Given the exploratory nature of this cross-sectional study, our aim was to gain initial insights into the communication preferences of Romanian ENT cancer patients. While confirmatory factor analysis (CFA) could provide further validation for the KOPRA scale, our primary focus was on investigating the relationship between communication preferences and psychological distress in this specific population. CFA was not performed, as it typically requires a larger sample size and more extensive resources. Future research could employ CFA to confirm the factor structure of the KOPRA scale in a larger and more diverse sample.

Finally, this study did not include the perspectives of healthcare providers, which could offer valuable insights into the challenges and opportunities for improving communication in oncology care. Future research should address these limitations by employing multi-center designs, incorporating diverse data collection methods, and including the perspectives of both patients and healthcare providers.

## 5. Conclusions

This study investigated the communication preferences and psychological distress of Romanian adults diagnosed with ENT cancers. Patients strongly prioritize active involvement in their care and open communication with healthcare providers. While emotional support is valued, clear information and active participation are prioritized over discussions about personal circumstances. Notably, this study revealed a high prevalence of psychological distress, particularly among widowed individuals and females. Healthcare providers should be sensitive to these vulnerabilities and tailor their communication and support strategies accordingly. Although no direct correlation was found between communication preferences and distress, this study underscores the complex interplay of these factors and the need for personalized approaches to cancer care that address both the informational and emotional needs of patients.

## Figures and Tables

**Table 1:** General characteristics of study population.

Age, mean (SD)	64.29 (10.9)
Sex, *n* (%) Male Female	109 (70.3) 46 (29.7)
Marital status, *n* (%) Married or living with partner Divorced Single Widowed	69 (44.5) 33 (21.3) 31 (20) 21 (13.5)
Qualifications, *n* (%) Primary school High school or professional school University Postgraduate studies	39 (25.2) 74 (47.7) 41 (26.5) 1 (0.6)
Occupation, *n* (%) Employed Retired Welfare Unemployed	58 (37.4) 70 (45.2) 16 (10.3) 11 (7.1)
Disease status, *n* (%) Recent diagnosis (less than 3 months) Progression Relapse Unknown	116 (74.8) 22 (14.2) 14 (9) 3 (1.9)
Area of residence, *n* (%) Urban Rural	79 (51) 76 (49)

**Table 2:** The KOPRA questionnaire.

KOPRA Items		Mean (SD)
Patient Participation and Orientation (PPO)	Your doctor should: weigh the advantages and disadvantages of different treatment options with you set treatment and therapy measures in a joint discussion with you discuss the treatment plan with you explain the procedure of your treatment to you thoroughly ask you what helped you in your treatment and what did not discuss the next stage of treatment with you ask you everything about your illness explain the procedure for your treatment summarize the results at the end of a discussion with you ask you how you assess the results of treatment sometimes address personal issues related to your illness	1. 4.58 (0.63) 2. 4.39 (0.71) 3. 4.44 (0.65) 4. 4.35 (0.74) 5. 4.45 (0.68) 6. 4.30 (0.78) 7. 4.37 (0.71) 8. 4.44 (0.68) 9. 4.31 (0.71) 10. 4.23 (0.95) 11. 4.24 (0.81)
Effective and Open Communication (EOC)	Your doctor should: 12. listen carefully when you want to say something 13. inform you at the end of treatment about the further treatment of your illness 14. inform you openly and directly of things concerning your illness that could be stressful (e.g., side effects of a treatment) 15. ask about all your symptoms 16. always tell you everything about your illness, even if it is unpleasant 17. ask you at the beginning of treatment to discuss all of your symptoms in detail 18. enable you to ask questions 19. ask you what you want to know about your treatment 20. ask whether you experience pain during therapy/treatment 21. explain to you exactly what your diagnosis means	12. 4.23 (0.77) 13. 4.24 (0.74) 14. 4.24 (0.77) 15. 4.24 (0.73) 16. 4.20 (0.77) 17. 4.27 (0.76) 18. 4.25 (0.71) 19. 4.25 (0.66) 20. 4.24 (0.70) 21. 4.27 (0.65)
Emotionally Supportive Communication (ESC)	Your doctor should: 22. always be optimistic and upbeat during talks with you 23. give you encouragement during talks 24. always be very even-tempered during talks 25. sometimes laugh when talking with you 26. exude calm during talks 27. always greet you warmly	22. 3.30 (0.68) 23. 3.33 (0.58) 24. 3.32 (0.57) 25. 3.20 (0.67) 26. 3.24 (0.62) 27. 2.71 (0.73)
Communication about Personal Circumstances (CPC)	Your doctor should: 28. sometimes speak with you on a personal level 29. sometimes talk with you about things that have nothing to do with your illness 30. occasionally talk to you about private matters 31. ask about your personal circumstances in order to find our something about you 32. try to develop a personal relationship with you	28. 2.08 (0.29) 29. 2.11 (0.30) 30. 2.08 (0.27) 31. 2.05 (0.20) 32. 2.43 (0.34)

**Table 3:** Psychological distress levels.

Variable	Mean (SD)	Patients with Scores 8–10; *n* (%)	Patients with Scores ≥ 11; *n* (%)
HADS–Depression	9.19 (2.7)	56 (36.12%)	49 (31.61%)
HADS–Anxiety	10.28 (2.8)	48 (30.96%)	70 (45.16%)

**Table 4:** Association between sociodemographic factors and anxiety/depression.

Sociodemographic Factor	HADS Subscale	*p*-Value	Significant Difference/Correlation
Age	Depression Anxiety	0.095 0.480	No No
Sex	Depression Anxiety	0.055 0.045	Marginally significant, females higher Significant, females higher
Marital Status	Depression Anxiety	0.037 0.036	Significant, widowed higher than married/cohabiting Significant, widowed higher than divorced and married/cohabiting

## Data Availability

Data are available upon request from the corresponding author.

## References

[1] King A., Hoppe R.B. (2013). “Best practice” for patient-centered communication: A narrative review. J. Grad. Med. Educ..

[2] Ellis P.M., Tattersall M.H.N. (1999). How should doctors communicate the diagnosis of cancer to patients?. Ann. Med..

[3] Parker P.A., Baile W.F., de Moor C., Lenzi R., Kudelka A.P., Cohen L. (2001). Breaking bad news about cancer: Patients’ preferences for communication. J. Clin. Oncol..

[4] Chen M., Wu V.S., Falk D., Cheatham C., Cullen J., Hoehn R. (2024). Patient Navigation in Cancer Treatment: A Systematic Review. Curr. Oncol. Rep..

[5] Fallowfield L., Jenkins V. (1999). Effective communication skills are the key to good cancer care. Eur. J. Cancer.

[6] Beck R.S., Daughtridge R., Sloane P.D. (2002). Physician-patient communication in the primary care office: A systematic review. J. Am. Board. Fam. Pract..

[7] Tuckett A.G. (2004). Truth-telling in clinical practice and the arguments for and against: A review of the literature. Nurs. Ethic..

[8] Mast M.S., Kindlimann A., Langewitz W. (2005). Recipients’ perspective on breaking bad news: How you put it really makes a difference. Patient Educ. Couns..

[9] Vicente R.S., Freitas A.R., Ferreira R.M.A., Prada S.P., Martins T.C., Mendes A.D., Vitorino M.M., Chaves A.F., Santos C.C., Costa D.A. (2023). Communication preferences and perceptions of cancer patient during their first medical oncology appointment. Psychooncology.

[10] Kim S., Kim J., Shim E., Hahm B., Yu E. (2020). Patients’ communication preferences for receiving a cancer diagnosis: Differences depending on cancer stage. Psychooncology.

[11] Krieger T., Salm S., Dresen A., Cecon N. (2022). Cancer patients’ experiences and preferences when receiving bad news: A qualitative study. J. Cancer Res. Clin. Oncol..

[12] Daskivich T.J., Gale R., Luu M., Khodyakov D., Anger J.T., Freedland S.J., Spiegel B. (2021). Patient Preferences for Communication of Life Expectancy in Prostate Cancer Treatment Consultations. JAMA Surg..

[13] Back A.L. (2020). Patient-Clinician Communication Issues in Palliative Care for Patients with Advanced Cancer. J. Clin. Oncol..

[14] Ryan R.E., Connolly M., Bradford N.K., Henderson S., Herbert A., Schonfeld L., Young J., Bothroyd J.I., Henderson A. (2022). Interventions for interpersonal communication about end of life care between health practitioners and affected people. Cochrane Database Syst. Rev..

[15] Ramchandani A., Mihic-Góngora L., Hernández R., Zafra-Poves M., Muñoz M.M., Ferreira E., Cruz-Castellanos P., Fernández-Montes A., Pacheco-Barcia V., Jiménez-Fonseca P. (2023). Psychological factors and prognostic communication preferences in advanced cancer: Multicentre study. BMJ Support. Palliat. Care.

[16] Farin E., Gramm L., Kosiol D. (2011). Development of a questionnaire to assess communication preferences of patients with chronic illness. Patient Educ. Couns..

[17] Ullrich A., Hauer J., Farin E. (2014). Communication preferences in patients with fibromyalgia syndrome: Descriptive results and patient characteristics as predictors. Patient Prefer. Adherence.

[18] Schmidt E., Gramm L., Farin E. (2012). Kommunikationspräferenzen chronischer Rückenschmerzpatienten in der medizinischen Rehabilitation [Communication preferences of patients with chronic back pain in medical rehabilitation]. Der Schmerz.

[19] Zigmond A.S., Snaith R.P. (1983). The hospital anxiety and depression scale. Acta Psychiatr. Scand..

[20] Bjelland I., Dahl A.A., Haug T.T., Neckelmann D. (2002). The validity of the Hospital Anxiety and Depression Scale. An updated literature review. J. Psychosom. Res..

[21] Razavi D., Delvaux N., Farvacques C., Robaye E. (1990). Screening for psychological distress, anxiety and depression in head and neck cancer patients: Performance of the Hospital Anxiety and Depression Scale (HADS). Head Neck.

[22] Roter D.L., Hall J.A., Aoki Y. (2002). Physician gender effects in medical communication. JAMA.

[23] Roter D.L., Hall J.A. (2004). Physician gender and patient-centered communication: A critical review of empirical research. Annu. Rev. Public Health.

[24] McKinstry B. (2000). Do patients wish to be involved in decision making in the consultation? A cross sectional survey with video vignettes. BMJ.

[25] Kennifer S.L., Alexander S.C., Pollak K.I., Jeffreys A.S., Olsen M.K., Rodriguez K.L., Arnold R.M., Tulsky J.A. (2009). Negative emotions in cancer care: Do oncologist’ responses depend on severity and type of emotion?. Patient Educ. Couns..

[26] Hack T.F., Degner L.F., Parker P.A. (2005). The communication goals and needs of cancer patients: A review. Psycho-Oncol..

[27] Street R.L., Makoul G., Arora N.K., Epstein R.M. (2009). How does communication heal? Pathways linking clinician–patient communication to health outcomes. Patient Educ. Couns..

[28] Arora N.K., McHorney C.A. (2000). Patient preferences for medical decision making. Med. Care.

[29] Yoshida S., Shimizu K., Matsui M., Fujimori M., Uchitomi Y., Horibe K. (2023). Preferred Communication with Adolescent and Young Adult Patients Receiving Bad News About Cancer. J. Adolesc. Young Adult Oncol..

[30] Gu L., Deng J., Xu H., Zhang S., Gao M., Qu Z., Zhang W., Tian D. (2019). The impact of contract service policy and doctor communication skills on rural patient-doctor trust relationship in the village clinics of three counties. BMC Health Serv. Res..

[31] Eadie T.L., Lamvik K., Baylor C.R., Yorkston K.M., Kim J., Amtmann D. (2014). Communicative participation and quality of life in head and neck cancer. Ann. Otol. Rhinol. Laryngol..

[32] Sauder C., Kapsner-Smith M., Baylor C., Yorkston K., Futran N., Eadie T. (2020). Communicative Participation and Quality of Life in Pretreatment Oral and Oropharyngeal Head and Neck Cancer. Otolaryngol. Neck Surg..

